# 2-Chloro-*N*′-[(*E*)-(2-meth­oxy-1-naphth­yl)methyl­ene]benzohydrazide

**DOI:** 10.1107/S1600536809016936

**Published:** 2009-05-14

**Authors:** Chunbao Tang

**Affiliations:** a Department of Chemistry, Jiaying University, Meizhou 514015, People’s Republic of China

## Abstract

In the mol­ecule of the title Schiff base compound, C_19_H_15_ClN_2_O_2_, the dihedral angle between the benzene ring and naphthyl ring system is 77.1 (2)°. In the crystal structure, centrosymmetrically related mol­ecules are linked into dimers through pairs of inter­molecular N–H⋯O hydrogen bonds, generating rings of graph set *R* ^2^_2_(8).

## Related literature

For related structures, see: Tang (2007 ▶, 2008 ▶). For bond-length data, see: Allen *et al.* (1987 ▶). For graph-set analysis, see: Etter *et al.* (1990 ▶); Bernstein *et al.* (1995 ▶).
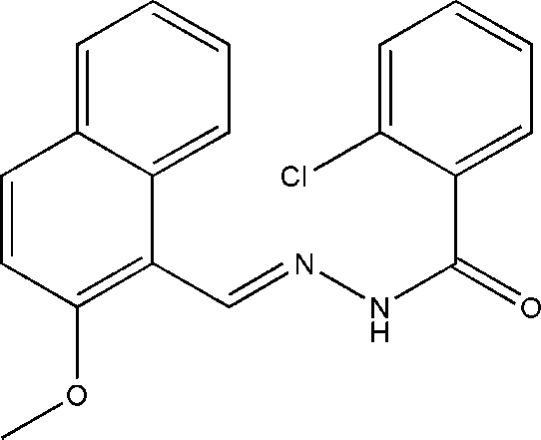


## Experimental

### 

#### Crystal data

C_19_H_15_ClN_2_O_2_
*M* *_r_* = 338.78
Monoclinic, 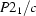

*a* = 10.751 (2) Å
*b* = 11.405 (2) Å
*c* = 14.376 (3) Å
β = 107.794 (10)°
*V* = 1678.4 (6) Å^3^
*Z* = 4
Mo *K*α radiation
μ = 0.24 mm^−1^
*T* = 298 K
0.30 × 0.28 × 0.27 mm

#### Data collection

Bruker SMART CCD area-detector diffractometer
Absorption correction: multi-scan (*SADABS*; Sheldrick, 1996 ▶) *T*_min_ = 0.931, *T*_max_ = 0.938
13186 measured reflections
3473 independent reflections
1295 reflections with *I* > 2σ(*I*)
*R*_int_ = 0.158

#### Refinement

*R*[*F* ^2^ > 2σ(*F* ^2^)] = 0.062
*wR*(*F* ^2^) = 0.142
*S* = 0.88
3473 reflections
221 parameters
1 restraint
H atoms treated by a mixture of independent and constrained refinement
Δρ_max_ = 0.22 e Å^−3^
Δρ_min_ = −0.20 e Å^−3^

### 

Data collection: *SMART* (Bruker, 2002 ▶); cell refinement: *SAINT* (Bruker, 2002 ▶); data reduction: *SAINT*; program(s) used to solve structure: *SHELXS97* (Sheldrick, 2008 ▶); program(s) used to refine structure: *SHELXL97* (Sheldrick, 2008 ▶); molecular graphics: *SHELXTL* (Sheldrick, 2008 ▶); software used to prepare material for publication: *SHELXL97*.

## Supplementary Material

Crystal structure: contains datablocks global, I. DOI: 10.1107/S1600536809016936/rz2320sup1.cif

Structure factors: contains datablocks I. DOI: 10.1107/S1600536809016936/rz2320Isup2.hkl

Additional supplementary materials:  crystallographic information; 3D view; checkCIF report

## Figures and Tables

**Table 1:** Hydrogen-bond geometry (Å, °)

*D*—H⋯*A*	*D*—H	H⋯*A*	*D*⋯*A*	*D*—H⋯*A*
N2—H2⋯O2^i^	0.90 (3)	1.99 (3)	2.886 (4)	172 (4)

Symmetry code: (i) 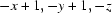.

**Fig. 1.**
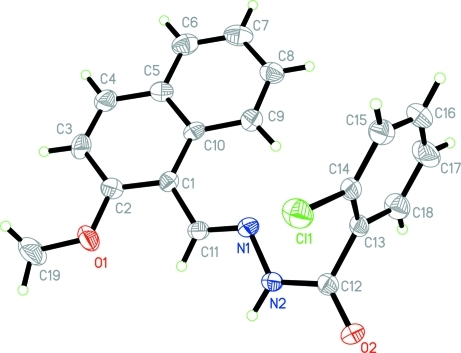
The molecular structure of the title compound, showing the atom-numbering scheme. Displacement ellipsoids are drawn at the 30% probability level.

**Fig. 2.**
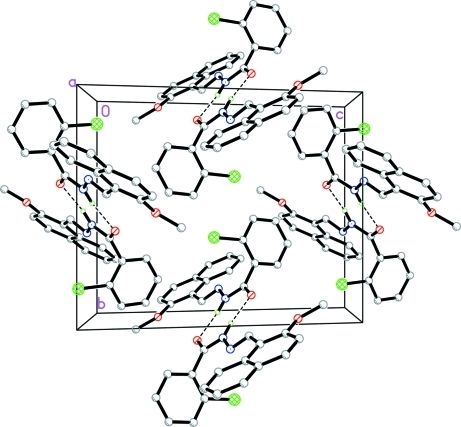
Packing diagram of the title compound viewed along the a axis. Intermolecular hydrogen bonds are drawn as dashed lines. Hydrogen atoms not involved in hydrogen bonding are omitted for clarity.

## supplementary crystallographic information

### Comment

Recently, the author has reported the structures of a few Schiff base compounds (Tang, 2007; Tang 2008). In a continuation of work in this area, the crystal structure of the title compound is reported herein.

In the title compound (Fig. 1), the dihedral angle between the benzene ring and the naphthyl ring system is 77.1 (2) °. The molecule adopts an E configuration about the C═N bond. All the bond lengths are within normal values (Allen *et al.*, 1987). In the crystal structure (Fig. 2), centrosymmetrically related molecules are linked into dimers through intermolecular N–H···O hydrogen bonds (Table 1), forming rings of graph set *R*^2^_2_(8) (Etter *et al.*, 1990; Bernstein *et al.*, 1995).

### Experimental

2-Methoxy-1-naphthylaldehyde (0.1 mmol, 18.6 mg) and 2-chlorobenzohydrazide (0.1 mmol, 12.6 mg) were dissolved in a methanol solution (20 ml). The mixture was stirred at reflux for 10 min to give a clear colourless solution. Colourless block-like crystals of the compound were formed by slow evaporation of the solvent over several days.

### Refinement

Atom H2 was located from a difference Fourier map and refined isotropically, with *U*_iso_ restrained to 0.08Å^2^. Other H atoms were constrained to ideal geometries, with C–H = 0.93–0.96 Å, and with *U*_iso_(H) = 1.2*U*_eq_(C) or 1.5*U*_eq_(C) for methyl H atoms.

### Crystal data

**Table:**

C_19_H_15_ClN_2_O_2_	*F*(000) = 704
*M**_r_* = 338.78	*D*_x_ = 1.341 Mg m^−^^3^
Monoclinic, *P*2_1_/*c*	Mo *K*α radiation, λ = 0.71073 Å
Hall symbol: -P 2ybc	Cell parameters from 394 reflections
*a* = 10.751 (2) Å	θ = 2.3–24.5°
*b* = 11.405 (2) Å	µ = 0.24 mm^−^^1^
*c* = 14.376 (3) Å	*T* = 298 K
β = 107.794 (10)°	Block, colourless
*V* = 1678.4 (6) Å^3^	0.30 × 0.28 × 0.27 mm
*Z* = 4	

### Data collection

**Table:**

Bruker SMART CCD area-detector diffractometer	3473 independent reflections
Radiation source: fine-focus sealed tube	1295 reflections with *I* > 2σ(*I*)
graphite	*R*_int_ = 0.158
ω scans	θ_max_ = 26.5°, θ_min_ = 2.0°
Absorption correction: multi-scan (*SADABS*; Sheldrick, 1996)	*h* = −13→12
*T*_min_ = 0.931, *T*_max_ = 0.938	*k* = −14→14
13186 measured reflections	*l* = −18→17

### Refinement

**Table:**

Refinement on *F*^2^	Primary atom site location: structure-invariant direct methods
Least-squares matrix: full	Secondary atom site location: difference Fourier map
*R*[*F*^2^ > 2σ(*F*^2^)] = 0.062	Hydrogen site location: inferred from neighbouring sites
*wR*(*F*^2^) = 0.142	H atoms treated by a mixture of independent and constrained refinement
*S* = 0.88	*w* = 1/[σ^2^(*F*_o_^2^) + (0.0423*P*)^2^] where *P* = (*F*_o_^2^ + 2*F*_c_^2^)/3
3473 reflections	(Δ/σ)_max_ < 0.001
221 parameters	Δρ_max_ = 0.22 e Å^−^^3^
1 restraint	Δρ_min_ = −0.20 e Å^−^^3^

### Special details

**Table:**

Geometry. All esds (except the esd in the dihedral angle between two l.s. planes) are estimated using the full covariance matrix. The cell esds are taken into account individually in the estimation of esds in distances, angles and torsion angles; correlations between esds in cell parameters are only used when they are defined by crystal symmetry. An approximate (isotropic) treatment of cell esds is used for estimating esds involving l.s. planes.
Refinement. Refinement of F^2^ against ALL reflections. The weighted R-factor wR and goodness of fit S are based on F^2^, conventional R-factors R are based on F, with F set to zero for negative F^2^. The threshold expression of F^2^ > 2sigma(F^2^) is used only for calculating R-factors(gt) etc. and is not relevant to the choice of reflections for refinement. R-factors based on F^2^ are statistically about twice as large as those based on F, and R- factors based on ALL data will be even larger.

### Fractional atomic coordinates and isotropic or equivalent isotropic displacement parameters (Å^2^)

**Table:**

	*x*	*y*	*z*	*U*_iso_*/*U*_eq_	
Cl1	0.58262 (12)	0.13770 (9)	0.03814 (8)	0.0647 (4)	
N2	0.6442 (3)	0.4212 (3)	−0.0046 (2)	0.0427 (8)	
O1	0.9807 (3)	0.5231 (3)	0.2634 (2)	0.0675 (9)	
N1	0.7735 (3)	0.3824 (3)	0.0222 (2)	0.0435 (9)	
C1	0.9913 (4)	0.4199 (3)	0.1258 (3)	0.0386 (10)	
O2	0.4392 (3)	0.3960 (2)	−0.10180 (18)	0.0516 (8)	
C10	1.0644 (4)	0.3584 (3)	0.0738 (3)	0.0397 (10)	
C12	0.5539 (4)	0.3633 (3)	−0.0752 (3)	0.0416 (10)	
C13	0.5987 (3)	0.2590 (3)	−0.1185 (3)	0.0379 (10)	
C2	1.0565 (4)	0.4677 (3)	0.2165 (3)	0.0474 (11)	
C11	0.8515 (4)	0.4435 (3)	0.0891 (3)	0.0413 (10)	
H11	0.8179	0.5057	0.1158	0.050*	
C5	1.2017 (4)	0.3485 (3)	0.1163 (3)	0.0493 (11)	
C14	0.6132 (3)	0.1509 (4)	−0.0725 (3)	0.0417 (10)	
C6	1.2771 (4)	0.2898 (4)	0.0656 (4)	0.0627 (13)	
H6	1.3670	0.2834	0.0940	0.075*	
C9	1.0092 (4)	0.3089 (3)	−0.0203 (3)	0.0480 (11)	
H9	0.9197	0.3150	−0.0509	0.058*	
C3	1.1932 (4)	0.4552 (4)	0.2586 (3)	0.0581 (12)	
H3	1.2352	0.4869	0.3198	0.070*	
C8	1.0851 (4)	0.2527 (3)	−0.0668 (3)	0.0547 (12)	
H8	1.0462	0.2203	−0.1282	0.066*	
C15	0.6539 (4)	0.0537 (4)	−0.1128 (3)	0.0568 (12)	
H15	0.6640	−0.0183	−0.0810	0.068*	
C7	1.2201 (5)	0.2428 (4)	−0.0240 (4)	0.0675 (14)	
H7	1.2705	0.2042	−0.0567	0.081*	
C18	0.6229 (4)	0.2670 (4)	−0.2066 (3)	0.0590 (13)	
H18	0.6119	0.3385	−0.2392	0.071*	
C4	1.2617 (4)	0.3971 (4)	0.2094 (3)	0.0604 (13)	
H4	1.3514	0.3886	0.2375	0.072*	
C16	0.6793 (4)	0.0650 (4)	−0.2006 (3)	0.0675 (14)	
H16	0.7074	0.0004	−0.2281	0.081*	
C17	0.6633 (5)	0.1709 (5)	−0.2476 (3)	0.0726 (15)	
H17	0.6798	0.1779	−0.3072	0.087*	
C19	1.0386 (5)	0.5842 (4)	0.3518 (4)	0.1002 (19)	
H19A	1.0830	0.5296	0.4017	0.150*	
H19B	0.9720	0.6240	0.3714	0.150*	
H19C	1.1001	0.6404	0.3423	0.150*	
H2	0.620 (4)	0.483 (2)	0.025 (3)	0.080*	

### Atomic displacement parameters (Å^2^)

**Table:**

	*U*^11^	*U*^22^	*U*^33^	*U*^12^	*U*^13^	*U*^23^
Cl1	0.0873 (9)	0.0637 (8)	0.0513 (7)	0.0114 (7)	0.0332 (6)	0.0104 (6)
N2	0.035 (2)	0.045 (2)	0.047 (2)	0.0046 (19)	0.0109 (17)	−0.0093 (18)
O1	0.061 (2)	0.085 (2)	0.0540 (19)	0.0004 (18)	0.0137 (17)	−0.0316 (18)
N1	0.032 (2)	0.048 (2)	0.048 (2)	0.0042 (17)	0.0085 (18)	−0.0042 (18)
C1	0.036 (3)	0.035 (2)	0.041 (3)	−0.003 (2)	0.007 (2)	0.001 (2)
O2	0.0356 (18)	0.0600 (19)	0.0539 (18)	0.0118 (15)	0.0059 (15)	−0.0056 (15)
C10	0.030 (2)	0.045 (3)	0.045 (3)	0.005 (2)	0.012 (2)	0.009 (2)
C12	0.041 (3)	0.048 (3)	0.037 (2)	0.001 (2)	0.015 (2)	−0.003 (2)
C13	0.036 (3)	0.052 (3)	0.028 (2)	0.000 (2)	0.013 (2)	−0.003 (2)
C2	0.046 (3)	0.045 (3)	0.053 (3)	0.000 (2)	0.018 (2)	−0.003 (2)
C11	0.040 (3)	0.040 (3)	0.044 (3)	0.006 (2)	0.014 (2)	0.001 (2)
C5	0.049 (3)	0.043 (3)	0.058 (3)	0.003 (2)	0.020 (3)	0.003 (2)
C14	0.039 (3)	0.052 (3)	0.034 (2)	0.003 (2)	0.012 (2)	−0.004 (2)
C6	0.044 (3)	0.058 (3)	0.084 (4)	0.003 (3)	0.017 (3)	0.003 (3)
C9	0.044 (3)	0.052 (3)	0.048 (3)	−0.002 (2)	0.015 (2)	0.003 (2)
C3	0.045 (3)	0.064 (3)	0.052 (3)	−0.007 (2)	−0.004 (2)	−0.011 (3)
C8	0.064 (4)	0.051 (3)	0.055 (3)	0.008 (2)	0.027 (3)	0.004 (2)
C15	0.063 (3)	0.053 (3)	0.053 (3)	0.007 (2)	0.016 (2)	−0.001 (3)
C7	0.059 (4)	0.064 (3)	0.091 (4)	0.010 (3)	0.040 (3)	0.000 (3)
C18	0.077 (4)	0.058 (3)	0.046 (3)	0.005 (3)	0.024 (3)	0.005 (2)
C4	0.045 (3)	0.061 (3)	0.066 (3)	0.004 (2)	0.003 (3)	−0.009 (3)
C16	0.073 (3)	0.069 (4)	0.062 (3)	0.012 (3)	0.022 (3)	−0.022 (3)
C17	0.097 (4)	0.081 (4)	0.051 (3)	0.015 (3)	0.038 (3)	−0.009 (3)
C19	0.091 (4)	0.105 (4)	0.097 (4)	0.004 (3)	0.018 (3)	−0.064 (4)

### Geometric parameters (Å, °)

**Table:**

Cl1—C14	1.726 (4)	C6—C7	1.356 (6)
N2—C12	1.344 (5)	C6—H6	0.9300
N2—N1	1.395 (4)	C9—C8	1.362 (5)
N2—H2	0.90 (3)	C9—H9	0.9300
O1—C2	1.361 (4)	C3—C4	1.342 (5)
O1—C19	1.416 (4)	C3—H3	0.9300
N1—C11	1.273 (4)	C8—C7	1.398 (6)
C1—C2	1.390 (5)	C8—H8	0.9300
C1—C10	1.424 (5)	C15—C16	1.377 (5)
C1—C11	1.458 (5)	C15—H15	0.9300
O2—C12	1.232 (4)	C7—H7	0.9300
C10—C9	1.418 (5)	C18—C17	1.375 (5)
C10—C5	1.419 (5)	C18—H18	0.9300
C12—C13	1.490 (5)	C4—H4	0.9300
C13—C18	1.371 (5)	C16—C17	1.369 (6)
C13—C14	1.385 (5)	C16—H16	0.9300
C2—C3	1.415 (5)	C17—H17	0.9300
C11—H11	0.9300	C19—H19A	0.9600
C5—C4	1.409 (5)	C19—H19B	0.9600
C5—C6	1.413 (5)	C19—H19C	0.9600
C14—C15	1.382 (5)		
			
C12—N2—N1	118.8 (3)	C8—C9—H9	119.5
C12—N2—H2	119 (3)	C10—C9—H9	119.5
N1—N2—H2	122 (3)	C4—C3—C2	119.3 (4)
C2—O1—C19	120.5 (3)	C4—C3—H3	120.3
C11—N1—N2	114.0 (3)	C2—C3—H3	120.3
C2—C1—C10	119.0 (4)	C9—C8—C7	121.3 (4)
C2—C1—C11	115.8 (4)	C9—C8—H8	119.4
C10—C1—C11	125.1 (4)	C7—C8—H8	119.4
C9—C10—C5	117.1 (4)	C16—C15—C14	119.1 (4)
C9—C10—C1	124.0 (4)	C16—C15—H15	120.4
C5—C10—C1	118.8 (4)	C14—C15—H15	120.4
O2—C12—N2	120.6 (4)	C6—C7—C8	119.6 (4)
O2—C12—C13	122.2 (4)	C6—C7—H7	120.2
N2—C12—C13	117.2 (4)	C8—C7—H7	120.2
C18—C13—C14	118.4 (4)	C13—C18—C17	121.1 (4)
C18—C13—C12	120.7 (4)	C13—C18—H18	119.5
C14—C13—C12	120.9 (3)	C17—C18—H18	119.5
O1—C2—C1	116.1 (4)	C3—C4—C5	122.0 (4)
O1—C2—C3	122.4 (4)	C3—C4—H4	119.0
C1—C2—C3	121.4 (4)	C5—C4—H4	119.0
N1—C11—C1	122.7 (4)	C17—C16—C15	120.3 (4)
N1—C11—H11	118.6	C17—C16—H16	119.8
C1—C11—H11	118.6	C15—C16—H16	119.8
C4—C5—C6	120.5 (4)	C16—C17—C18	120.0 (4)
C4—C5—C10	119.4 (4)	C16—C17—H17	120.0
C6—C5—C10	120.1 (4)	C18—C17—H17	120.0
C15—C14—C13	121.1 (4)	O1—C19—H19A	109.5
C15—C14—Cl1	119.3 (3)	O1—C19—H19B	109.5
C13—C14—Cl1	119.5 (3)	H19A—C19—H19B	109.5
C7—C6—C5	120.9 (4)	O1—C19—H19C	109.5
C7—C6—H6	119.6	H19A—C19—H19C	109.5
C5—C6—H6	119.6	H19B—C19—H19C	109.5
C8—C9—C10	121.1 (4)		

### Hydrogen-bond geometry (Å, °)

**Table:**

*D*—H···*A*	*D*—H	H···*A*	*D*···*A*	*D*—H···*A*
N2—H2···O2^i^	0.90 (3)	1.99 (3)	2.886 (4)	172 (4)

Symmetry codes: (i) −*x*+1, −*y*+1, −*z*.
